# Clinical Spectrum of *SCN5A* Channelopathy in Children with Primary Electrical Disease and Structurally Normal Hearts

**DOI:** 10.3390/genes13010016

**Published:** 2021-12-22

**Authors:** Teresa Villarreal-Molina, Gabriela Paola García-Ordóñez, Álvaro E. Reyes-Quintero, Mayra Domínguez-Pérez, Leonor Jacobo-Albavera, Santiago Nava, Alessandra Carnevale, Argelia Medeiros-Domingo, Pedro Iturralde

**Affiliations:** 1 Laboratory of Cardiovascular Genomics, National Institute of Genomic Medicine, Mexico City 14610, Mexico; mvillareal@inmegen.gob.mx (T.V.-M.); gabrielapgarciao12@gmail.com (G.P.G.-O.); mdominguez@inmegen.gob.mx (M.D.-P.); ljacobo@inmegen.gob.mx (L.J.-A.); 2 Departament of Electrocardiology, National Institute of Cardiology “Ignacio Chávez”, Mexico City 14080, Mexico; alvaroequintero@gmail.com (Á.E.R.-Q.); santiagonavat@hotmail.com (S.N.); 3 Laboratory of Mendelian Diseases, National Institute of Genomic Medicine, Mexico City 14610, Mexico; acarnevale@inmegen.gob.mx; 4 Swiss DNAlysis, Cardiogenetics, 8600 Dübendorf, Switzerland

**Keywords:** *SCN5A*-channelopathy, childhood onset, overlap syndrome, sick sinus syndrome, progressive cardiac conduction disease, Brugada syndrome, idiopathic ventricular tachycardia, long QT syndrome, compound heterozygosity

## Abstract

Sodium voltage-gated channel α subunit 5 (*SCN5A)*-mutations may cause an array of arrhythmogenic syndromes most frequently as an autosomal dominant trait, with incomplete penetrance, variable expressivity and male predominance. In the present study, we retrospectively describe a group of Mexican patients with *SCN5A*-disease causing variants in whom the onset of symptoms occurred in the pediatric age range. The study included 17 patients with clinical diagnosis of primary electrical disease, at least one *SCN5A* pathogenic or likely pathogenic mutation and age of onset <18 years, and all available first- and second-degree relatives. Fifteen patients (88.2%) were male, and sixteen independent variants were found (twelve missense, three truncating and one complex inframe deletion/insertion). The frequency of compound heterozygosity was remarkably high (3/17, 17.6%), with early childhood onset and severe disease. Overall, 70.6% of pediatric patients presented with overlap syndrome, 11.8% with isolated sick sinus syndrome, 11.8% with isolated Brugada syndrome (BrS) and 5.9% with isolated type 3 long QT syndrome (LQTS). A total of 24/45 *SCN5A* mutation carriers were affected (overall penetrance 53.3%), and penetrance was higher in males (63.3%, 19 affected/30 mutation carriers) than in females (33.3%, 5 affected/15 carriers). In conclusion, pediatric patients with *SCNA*-disease causing variants presented mainly as overlap syndrome, with predominant loss-of-function phenotypes of sick sinus syndrome (SSS), progressive cardiac conduction disease (PCCD) and ventricular arrhythmias.

## 1. Introduction

The function of the cardiac voltage-gated sodium channel α subunit protein (Na_V_1.5) is crucial because it initiates the cardiac action potential by generating the inward sodium current (I_Na_), which mediates cardiomyocyte excitability and impulse conduction in the myocardium and specialized conduction system. This channel also generates the late sodium current (I_NaL_), which plays a role in repolarization and refractoriness [1]. The α subunit of Na_V_1.5 is a 220 kDa transmembrane protein encoded by the sodium voltage-gated channel α subunit (*SCN5A*) gene, consisting of a cytoplasmic N-terminal domain, four homologous transmembrane domains (TMD, DI-DIV) and a cytoplasmic C-terminal domain. Each TMD has six transmembrane α-helix segments (S1–S6) connected by cytoplasmic linkers [2]. Although sodium channel α-subunits are traditionally believed to form functional monomers, some *SCN5A* mutation studies in patients with inherited arrhythmias indicate that oligomerization of the sodium channel α-subunits may occur, and recent experiments suggested that sodium channel α-subunits physically interact, assemble, function and gate as a dimer [3].

*SCN5A*-mutations may cause several arrhythmogenic phenotypes most frequently inherited in an autosomal dominant fashion [4,5]. These mutations cause an array of arrhythmogenic syndromes including dilated, arrhythmogenic or non-compaction cardiomyopathy, or arrhythmogenic syndromes with minimal or no structural defects such as sick sinus syndrome (SSS), progressive cardiac conduction disease (PCCD), type 1 Brugada syndrome (BrS), familial atrial fibrillation, idiopathic ventricular tachycardia (VT), multifocal ectopic Purkinje-related premature contractions (MEPPC), and type 3 long QT syndrome (LQTS) [6]. Characteristically, *SCN5A* disease-causing mutations show incomplete penetrance, variable expressivity and male predominance for reasons that are not fully understood, which include both genetic background and environmental factors [7].

Early age of onset is associated with severity in many genetic diseases. Although there are many isolated case-reports of *SCN5A*-channelopathy in children in the medical literature [8,9,10,11,12,13,14], few studies have analyzed *SCN5A*-channelopathy cohorts where onset of symptoms occurs in pediatric patients. A large prospective multi-center pediatric cohort of *SCN5A* mutation-positive neonates reported that 67.9% were asymptomatic at diagnosis, while age <1 year at diagnosis, compound heterozygous mutations, and mutations with both gain- and loss-of-function were identified as independent risk factors for cardiac events [15]. In the present study, we retrospectively describe a group of Mexican patients with *SCN5A*-disease causing variants in whom the onset of symptoms occurred in the pediatric age range, attending the Ignacio Chávez National Institute of Cardiology over a 15 year period. This study provides insight, which can potentially be of aid for diagnosis, phenotypic characterization and to define therapeutic approaches in pediatric patients with *SCN5A*-disease causing variants.

## 2. Materials and Methods

### 2.1. Subjects

The study included a group of pediatric patients (age of onset <18 years) recruited from the Department of Cardiac Electrophysiology at the National Institute of Cardiology “Ignacio Chavez” in Mexico City over a 15-year period (2005 to 2020). All patients had a clinical diagnosis of primary electrical disease (SSS, PCCD, BrS, VT, LQTS) and a sodium channel disease-causing variant. All available first- and second-degree relatives were also included in the study. Index cases underwent routine clinical evaluation, including interrogation of family and medical history, physical examination, electrocardiogram (standard and high precordial leads), 24-h Holter, stress test, and had a yearly clinical examination for follow-up. Structural heart disease was ruled out by *trans*-thoracic echocardiography and/or magnetic resonance imaging. Informed consent was provided by participants or by their parents or legal guardian. The study was approved by the Ethics Committees of the National Institute of Cardiology Ignacio Chávez and the National Institute of Genomic Medicine in Mexico City.

The arrhythmogenic phenotypes considered were the following: SSS, sinus bradycardia, sinus pause or arrest with or without escape rhythm, sinoatrial exit block, tachy-brady syndrome, atrial fibrillation with slow ventricular response in the absence of AV node blocking agents, chronotropic incompetence [16]; PCCD: finding of a major conduction defect on the electrocardiogram (ECG) such as complete right bundle branch block, complete left bundle branch block, left anterior fascicular block / hemiblock or left posterior hemiblock, prolonged PR interval or complete AV block with broad QRS complexes [17]; BrS, appearance of a type one ST-segment elevation (coved type) in more than one right precordial lead (V_1_ to V_3_), in the presence or absence of a sodium channel blocker, and one of the following: documented ventricular fibrillation, ventricular tachycardia or self-terminating polymorphic ventricular tachycardia, a family history of sudden cardiac death (SCD, <45 years), coved type ECGs in family members, electrophysiological inducibility, syncope, or nocturnal agonal breathing [18]; LQTS was diagnosed when considered as high probability according the criteria of Schwartz [19]. Patients with more than one of these arrhythmogenic phenotypes were considered as *SCN5A* overlap syndrome cases.

### 2.2. Targeted Sequencing

Genomic DNA was extracted from peripheral blood of all participants using commercial methods (QIAGEN DNA Midi blood kit^®^). Samples from all index cases were sequenced using the TruSight Cardio^®^ Sequencing Panel on a Mi-Seq device (Illumina, San Diego, CA, USA). Post-run sequencing quality was assessed with FastQC (Babraham Bioninformatics, UK), reads were aligned with Burrows-Wheeler v2.0 (Broad Institute, Cambridge, MA, USA) [20]; variant calling was performed with the Genome Analysis Tool Kit (GATK v4.0; https://gatk.broadinstitute.org; accessed on 8 September 2021), and all variants were annotated with ANNOVAR (http://annovar.openbioinformatics.org; accessed on 8 September 2021).

All novel or very low frequency variants (minor allele frequency <0.0005) affecting the amino acid sequence of arrhythmogenic channelopathy-related genes were classified according to the American College of Medical Genetics and Genomics criteria as benign, likely benign, of unknown clinical significance (VUS), likely pathogenic (LP) or pathogenic. Patients with at least one pathogenic or likely pathogenic *SCN5A* variant and their available relatives were included in the analysis. Amino acid numbering was made according to transcription variant NM_198056 (http://www.ncbi.nlm.nih.gov/, accessed on 8 September 2021).

### 2.3. Statistical Analysis

The numerical variables are expressed as mean ± SD or median and interquartile range (IQR) as appropriate, and categorical variables as percentages. Overall penetrance for arrhythmogenic syndromes was estimated including all available individuals from families in which at least one first-degree relative was screened, as the ratio between number of *SCN5A* mutation carriers with arrhythmogenic phenotypes (affected) and the total number of carriers (affected and non-affected). Missense variants found in compound heterozygosity in probands, with no symptomatic heterozygous carriers in the family, were excluded from the overall penetrance estimation. Differences in *SCN5A* mutation penetrance between males and females were compared using the Chi-squared test.

## 3. Results

### 3.1. SCN5A Mutations

A total of 17 apparently unrelated patients with at least one pathogenic or likely pathogenic *SCN5A* variant were included in the study, along with a total of 42 first- and second-degree relatives. Table 1 summarizes the characteristics of all pathogenic or likely pathogenic *SCN5A* variants identified in the probands [21,22,23,24,25,26,27,28,29]. A total of sixteen independent variants were identified, twelve were missense, while four were inferred as null (three truncating frameshift and one a complex inframe multiple amino acid (aa) deletion/insertion occurring in a non-repetitive aa sequence in DIII/S5 (p.Trp1345-Ser1349delinsPhe). All but three of the missense variants (p.Arg811Cys, p.Pro1730Leu and p.Ala1778Asp) had at least one functional study reporting loss and/or gain of function. Thirteen patients were heterozygous for missense, and one was heterozygous for a frameshift truncating mutation. Notably, three patients (17.6%) were compound heterozygous for an inferred null mutation and a missense *SCN5A* variant. Most missense mutations were located within the DIV domain (6/12, 50%), four (33.3%) affected the pore forming region (three in DIV and one in DII), while two affected voltage sensor domains (S4) in DII and DIV. No other pathogenic or likely pathogenic variants were found among 174 cardiogenes in this group of patients.

### 3.2. Clinical Characteristics of Pediatric Arrhythmogenic Channelopathy Patients Bearing SCN5A Mutations

**Figure 1:**
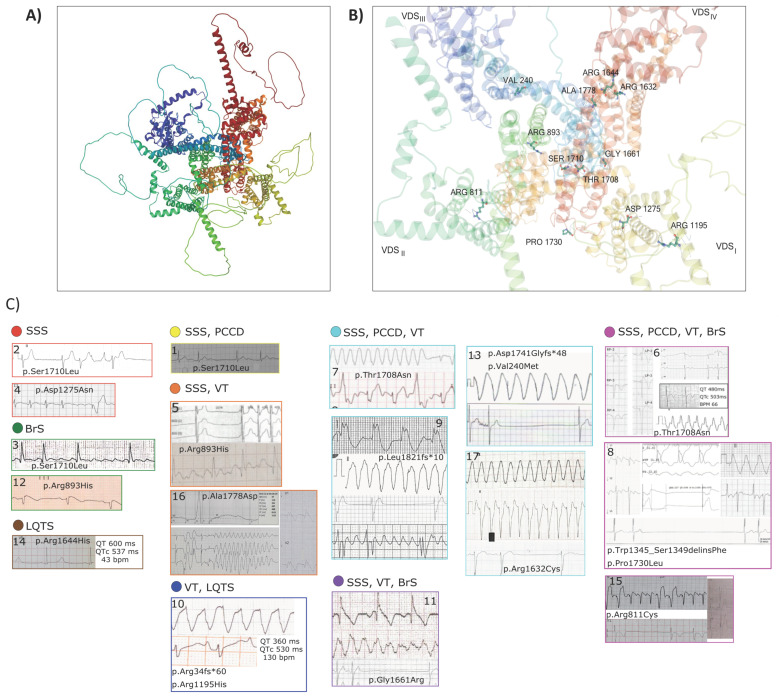
Molecular mapping of the *SCN5A* mutations. (**A**) Cartoon representation of the overall structure of Na_V_1.5: DI, DII, DIII and DIV domains are colored yellow, green, blue and red, respectively. (**B**) Upper view of the cryo-EM reconstruction of Na_V_1.5 using the UniProt (https://www.uniprot.org/uniprot/Q14524, accessed on 28 October 2021) and α Fold templates. *SCN5A* mutations are indicated as balls and sticks. (**C**) Representative ECGs of probands are presented. The case number is indicated on the ECG image. Cases are ordered according to arrhythmogenic phenotypes, SSS: sick sinus syndrome; PCCD: progressive cardiac conduction disease; VT: ventricular tachycardia; BrS: Brugada syndrome; LQTS: long QT syndrome. ECG from Case 2: atrial standstill; Case 4: atrial flutter with VVI pacemaker escape; Case 3: atrial flutter; Case 12: coved type ST elevation and sinus bradycardia; Case 14: sinus bradycardia with prolonged QTc interval (537 ms); Case 1: sinus bradycardia; Case 5: sinus standstill with monomorphic ventricular tachycardia; Case 16: sinus standstill with ventricular fibrillation; Case 10: monomorphic ventricular tachycardia; Case 7: sustained monomorphic ventricular tachycardia; Case 9: severe intraventricular conduction disease, monomorphic ventricular tachycardia, sinus standstill and atrial flutter; Case 13: sustained monomorphic ventricular tachycardia and sinus standstill; Case 17: monomorphic ventricular tachycardia and atrial standstill; Case 11: sinus bradycardia, ventricular fibrillation and sinus standstill; Case 6: sinus standstill, prolonged QTc interval (507ms) and sustained monomorphic ventricular tachycardia; Case 8: severe cardiac intraventricular conduction disease, sustained monomorphic ventricular tachycardia and sinus standstill; Case 15: sinus standstill and VVI pacemaker.

Demographic and clinical characteristics of all index cases are described in Table 2. Fifteen of these patients (88.2%) were male, and only two were female. Median age of symptoms onset was 6 years (IQR = 12 years). Onset of symptoms occurred in the early childhood range (<6 years) in eight patients (47.1%), during school age (6–12 years) in three patients (17.6%), and during teenage years in six patients (35.3%). Overall, the most prevalent arrhythmogenic syndrome was SSS (13/17, 76.5%), followed by VT (64.7%), PCCD (47.1%), type 1 BrS (35.3%) and LQTS (11.8%). Notably, twelve patients (70.6%) had overlap syndrome, two (11.8%) had isolated SSS, two more had isolated BrS, one (5.9%) had isolated type 3 LQTS. Considering only patients with overlap syndrome, the most prevalent phenotypes were SSS and VT (11/12, 91.7% each), followed by PCCD (66.7%), type 1 BrS (33.3%) and LQTS (8.3%). Pacemakers were implanted in all patients with SSS, and an implantable cardioverter defibrillator was implanted in the only patient who had suffered a ventricular fibrillation event. Figure 1 depicts mutation sites and ECGs from all probands.

Three apparently unrelated patients were heterozygous for the p.Ser1710Leu mutation, each with a different phenotype: overlap syndrome (SSS and PCCD), isolated SSS and isolated BrS. Two apparently unrelated patients were p.Arg893His heterozygous, one with overlap and the other with isolated BrS. In addition, two apparently unrelated children were heterozygous for the p.Thr1708Asn mutation, both with overlap syndrome and school-aged at the onset of symptoms. On further interrogation, family members were able to identify that both index cases were in fact second cousins once removed.

### 3.3. Syncope, Seizures, Non-Fatal Cardiac Arrest (NFCA) and Sudden Cardiac Death (SCD)

Syncope was reported in ten (58.8%) patients, and seizures in four (23.5%). The frequency of non-fatal cardiac arrest was 29.4% (5/17), and three of these five patients suffered SCD afterwards. The overall frequency of sudden cardiac death was also 29.4%, occurring before age 12 in 4 patients with overlap syndrome, and at age 17 in an isolated BrS patient.

### 3.4. ECG and Holter Findings

Twelve patients had at least one type of supraventricular tachycardia (SVT). The most prevalent was atrial flutter (8/17, 47.1%), followed by atrial fibrillation (35.3%), cavotricuspid isthmus-dependent atrial flutter (17.6%) and atrial tachycardia (11.8%). Moreover, twelve patients had one or more documented ventricular tachycardia events; the most prevalent was sustained monomorphic ventricular tachycardia (58.8%), followed by ventricular fibrillation (17.6%), polymorphic ventricular tachycardia (11.8%); and torsade de points (5.9%); ten had atrio-ventricular block (58.8%) and five had documented atrial standstill (29.4%). Finally, 15/17 patients (76.5%) showed a prolonged QTc interval (QTc max >460 ms), which was likely secondary to intraventricular conduction defect or AV block in 13, and only two patients had a normal maximum QTc interval (<440 ms).

### 3.5. Incomplete Penetrance and Variable Expressivity

**Figure 2:**
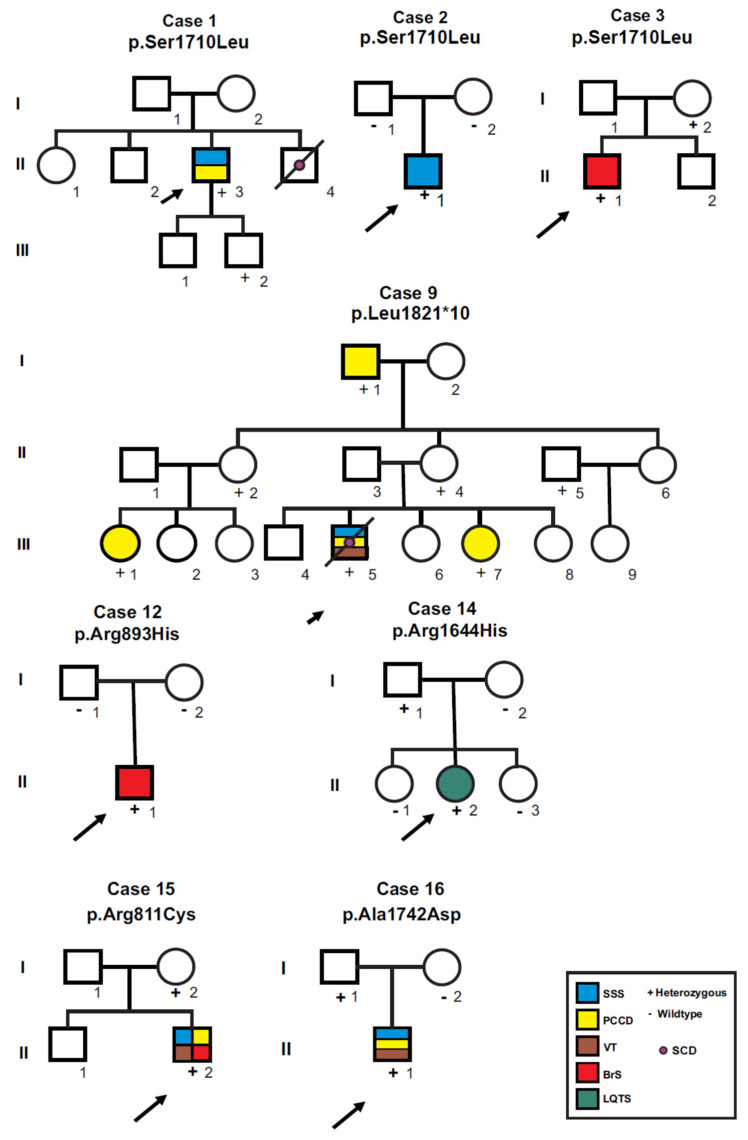
Pedigrees of probands heterozygous for *SCN5A* mutations where at least one first-degree relative was available for screening. Circles indicate females, squares, males; symbols with diagonal lines represent deceased individuals. Open symbols represent asymptomatic individuals, filled symbols were affected by arrhythmogenic syndromes according to the color code. Parentship was not confirmed by genetic analyses in any case. SSS: sick sinus syndrome; PCCD: progressive cardiac conduction disease; VT: ventricular tachycardia; BrS: Brugada syndrome; LQTS: long QT syndrome; SCD: sudden cardiac death.

**Figure 3:**
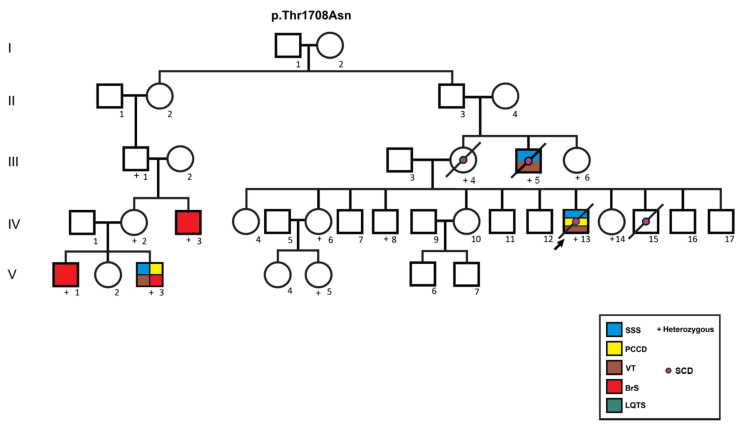
Pedigree of the family bearing the p.Thr1708Asn mutation. Circles indicate females, squares, males. Symbols with diagonal lines represent deceased individuals. Open symbols represent asymptomatic individuals, filled symbols are affected by arrhythmogenic syndromes according to the color code. Case 6 (V-3) and case 7 (IV-13) were initially thought to be unrelated, but after thorough interrogation were found to be first cousins once removed. Individual III-4 suffered sudden cardiac death at age 39 years, III-5 at age 56 years; and IV-15 at age 6 years. p.Thr1708Asn heterozygous individuals III-6, IV-6, IV-8, and V-5 had minor ECG manifestations, including prolonged PR and/or prolonged QTc intervals. SSS: sick sinus syndrome; PCCD: progressive cardiac conduction disease; VT: ventricular tachycardia; BrS: Brugada syndrome; LQTS: long QT syndrome; SCD: sudden cardiac death.

Figure 2 and Figure 3 show family pedigrees of index cases with autosomal dominant inheritance (heterozygous mutations), where at least one parent was available for clinical assessment and DNA analysis (all except cases 4, 5, 11 and 17), showing incomplete penetrance and variable expressivity. Different phenotypes were observed both for the same mutation in different cases (cases 1, 2 and 3 for p.Ser1710Leu; cases 6 and 7 for p.Thr1708Asn), and within the same family (pedigrees for cases 6, 7 and 9). Case 14 was the only isolated LQTS case, she and her father were heterozygous for the p.Arg1644His mutation; however, her father’s ECG showed a normal QTc interval (432 ms) with bradycardia (49 bpm). From a total of 45 *SCN5A* mutation carriers, 24 were affected with an arrhythmogenic phenotype (overall penetrance 53.3%), and penetrance was higher in males (63.3%, 19 affected/30 carriers) than in females (33.3%, five affected/15 carriers; *p* = 0.057).

Penetrance of individual mutations was estimated when five or more heterozygous carriers were found. A total of 14 individuals of a single kindred carried the p.Thr1708Asn mutation (eight male and six female); penetrance for arrhythmogenic phenotypes was 6/8 (75.0%) in males (three with overlap syndrome, two with BrS and one with sudden cardiac death at age 6). No arrhythmic phenotypes were observed in available female carriers, but 3/4 (75%) showed minor ECG abnormalities such as AV block (PR < 200 ms) and/or prolonged QTc interval (>470 ms, range 478–488 ms). Moreover, a total of seven individuals from a single kindred carried the p.Leu1821fs*10 mutation. Four were female (two with PCCD and two asymptomatic) and three were male (one with PCCD, one with overlap syndrome and one asymptomatic). Finally, five individuals from three different kindreds were heterozygous for the p.Ser1710Leu mutation, an asymptomatic female and four males (one asymptomatic, one each with overlap syndrome, PCCD and BrS).

### 3.6. Compound Heterozygosity for SCN5A Mutations

**Figure 4:**
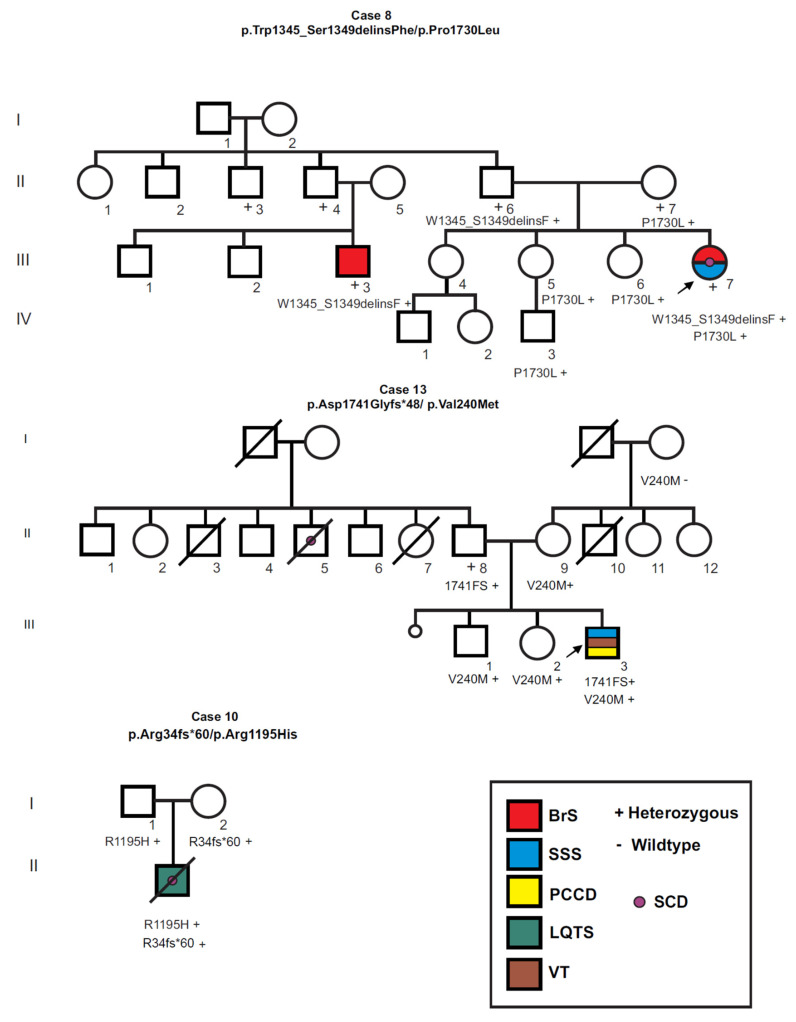
Pedigree of 3 overlap syndrome cases who were compound heterozygous for *SCN5A* mutations. Circles indicate females, squares, males; symbols with diagonal lines represent deceased individuals. Open symbols represent asymptomatic individuals, filled symbols were affected by arrhythmogenic syndromes according to the color code. SSS: sick sinus syndrome; PCCD: progressive cardiac conduction disease; VT: ventricular tachycardia; BrS: Brugada syndrome; LQTS: long QT syndrome; SCD: sudden cardiac death.

Figure 4 shows the pedigrees of the three index cases (17.6%) found to be compound heterozygous for *SCN5A* mutations, with an onset of symptoms in early childhood (younger than age 4 years), overlap syndrome and prolonged QTc intervals. Case 8 (p.Trp1345_Ser1349delinsPhe/ p.Pro1730Leu) was a severely affected six year-old girl who showed episodes of palpitations, fatigue and documented bradycardia since age three years, and was diagnosed with SSS, PCCD, VT, type 1 BrS and showed a prolonged QTc interval (495ms). During hospital stay she suffered various arrhythmic events including atrial flutter, atrial standstill, sustained ventricular tachycardia, ventricular fibrillation, and notably, bidirectional ventricular tachycardia and two cardiac arrest episodes with successful reanimation after cardio-pulmonary resuscitation. Both parents were asymptomatic, however a p.Trp1345_Ser1349delinsPhe heterozygous paternal first cousin showed a type 1 BrS ECG pattern. In addition, four asymptomatic relatives (II-7, III-5, III-6 and IV-3) carried the p.Pro1730Leu variant.

Case 10 (p.Arg34fs*60/p.Arg1195His) was a 22 month-old male toddler [27], who was admitted to the emergency room with febrile seizures and syncope and was diagnosed with SSS and VT, and a prolonged QTc interval (maximum QTc = 519 ms) with no apparent intraventricular conduction defect. The boy showed signs of severe neurological injury and died shortly after being admitted. He inherited the p.Arg1195His variant from his father, the p.Arg34fs*60 variant from his mother, and both parents were asymptomatic. Finally, case 13 (p.Asp1741Glyfs*48/p.Val240Met) presented with syncope at age 4 years, and was diagnosed with SSS, PCCD and recurrent monomorphic VT. The QT interval on ECG was prolonged (QTc = 630 ms). The mother (II-9) and two siblings (III-1 and III-2) had the Val240Met variant and were asymptomatic, while the father was p.Asp1741Glyfs*48 heterozygous and was also asymptomatic.

## 4. Discussion

The field of cardiovascular genetics is rapidly evolving and has greatly contributed to the understanding of primary electrical heart disease. However, this field faces an enormous challenge when it comes to the integration of genetics, functional studies, diagnosis, prognosis, and management [30]. The growing list of *SCN5A* channelopathy and overlap syndrome reports in the medical literature confirms that incomplete penetrance, male predominance and variable expressivity are characteristic of the disease, attributed to both genetic and non-genetic factors [7]. In the present study, all families with more than one *SCN5A* mutation carrier showed this variable expressivity, involving differences in age of onset (childhood–adulthood), arrhythmogenic phenotypes and disease severity. Similarly, apparently unrelated patients sharing the same mutation (Cases 1, 2 and 3 for p.Ser1710Leu) also showed variable expressivity regarding arrhythmogenic phenotypes. As expected, overall penetrance and penetrance of individual mutations was higher in male than in female individuals.

To our knowledge only one prospective study of children with *SCN5A* mutations included 442 neonates, where most showed no ECG alterations at birth (44.3%) and the most frequent arrhythmogenic phenotype was isolated PCCD (25.6%), followed by overlap syndrome (15%), LQTS (10.6%) and BrS (1.8%) [15]. The present study includes only pediatric patients who were symptomatic and required specialized medical assistance at the Ignacio Chávez National Institute of Cardiology. In agreement with the study in neonates, isolated LQTS and isolated BrS were the least frequent phenotypes (5.9% each), however among the symptomatic children of the present study, the most frequent phenotype was overlap syndrome (70.6%).

The high frequency of children with compound heterozygous *SCN5A* mutations in our group of symptomatic children (17.6%) is noteworthy. Compound heterozygosity is infrequent in *SCN5A* channelopathies, reported only in 13/2111 (0.62%) of BrS patients [31], and in 0.7% of neonates with *SCN5A* mutations [15]. Moreover, among ten individual *SCN5A* compound heterozygous case reports [12,13,27,32,33,34,35,36,37,38], only two cases reported adult age of onset [35,36], and 6/8 childhood onset cases had a truncating mutation combined with a missense mutation [12,27,32,33,34]. All three of our compound heterozygous cases (cases 8, 10 and 13) carried an inferred null and a missense mutation, had a severe overlap syndrome with early childhood onset (<4 years), and NFCA events occurred in two of these cases. Notably, case 8 had documented polymorphic ventricular tachycardia and bidirectional ventricular tachycardia, considered a calcium-handling arrhythmia. To our knowledge, there is only one report of a *SCN5A* mutation (p.T1857I) in a family with multiple sudden cardiac deaths where afflicted probands presented with atrial and ventricular arrhythmias including bidirectional VT [39]. Functional studies in heterologous cells and the use of O`Hara Rudy and Grandi models [40,41] suggested the mutation caused right-shifted voltage-dependence of both activation (gain of function, GOF) and inactivation (loss of function, LOF), with a net gain-of-function in the Na_V_1.5 gating, an increased window current and resultant ventricular tissue after depolarization.

Among the genetic modifiers contributing to variable expressivity and incomplete penetrance, studies in heterologous cells have shown common *SCN5A* polymorphisms p.H558R and p.del1077Q not only affect electrophysiological properties of Na_V_1.5, but may also modulate the effects of co-existing disease-causing mutations [42,43,44]. In this regard, a functional study in HEK293 cells showed that the p.Thr1708Asn mutation decreased the peak I_Na_ current and increased the late I_NaL_ current, decreasing the peak I_Na_ current even further when found in *cis* with the p.H558R polymorphism [25]. Segregation analysis of the p.Thr1708Asn kindred (Figure 3) showed that both variants were in fact on the same allele, as two of the p.Thr1708Asn carriers (IV-13 and IV-14) were p.558R homozygous (data not shown), compatible with the severe phenotype observed in several affected males. Unfortunately, the number of individuals with *SCN5A* mutations was insufficient to assess the possible association of common polymorphisms with clinical severity in our series.

The consequences of most of the mutations identified in this group of patients have been studied in heterologous ion channel expression systems [16,17,18,19,20,21,22,23,24]. However, because of the many consequences of mutations on electrophysiological phenotypes (early and late inward Na currents, activation or inactivation properties, interactions with other proteins, response to adrenergic or cholinergic stimuli, response to anti-arrhythmic drugs, etc.), and because different functional phenotypes often coexist, labeling variants as GOF and/or LOF may be an oversimplification. Particularly, because it has been proposed that Na_V_1.5 channels assemble as dimers [3], compound heterozygous mutations may require the biophysical characterization of both mutations separately and in co-expression. In this regard, previous functional studies of the mutations of case 10 showed the frame-shifted and prematurely truncated peptide *SCN5A*-Arg34fs*60 produced no current, while *SCN5A*-Arg1195His had a normal peak and late current but abnormal voltage-dependent gating parameters. Interestingly, co-expression of both variants led to a significant increase in late I_NaL_ current [27]. Functional studies of *SCN5A* variants found in case 8 (p.Trp1345_Ser1349delinsPhe/p.Pro1730Leu) and in case 13 (p.Asp1741Glyfs*48/ p.Val240Met), both individually and in co-expression, are pending, as are functional studies of three of the missense variants here identified (p.Arg811Cys; p.Pro1730Leu, p.Ala1778Asp). While these models have a number of shortcomings, as they do not closely reproduce human heart physiology and clinical manifestations [45,46], functional and pharmacological studies in induced pluripotent cell (iPSC)-derived cardiomyocytes have overcome several of these shortcomings and have become a promising option for precision medicine [47]. These functional studies, together with careful and detailed clinical characterization of patients and apparently asymptomatic mutation carriers, are crucial to understand the complexities of *SCN5A* channelopathies.

In conclusion, *SCN5A*-disease associated phenotypes occurring in the pediatric age were characterized mainly by overlap syndrome, sick sinus syndrome, cardiac conduction disease and ventricular tachycardia. It is important to recognize these phenotypes as potential markers of sodium channel disease, and to avoid the use of sodium channel blockers which can exacerbate the phenotype or even cause irreversible ventricular arrhythmias in the setting of pathogenic variants affecting *SCN5A*.

## Figures and Tables

**Table 1:** *SCN5A* variants identified in children with arrhythmogenic syndromes.

Variant	Variant Type	dbSNP	gnomAD Global MAF	Domain Location	Site	Functional Interpretation *	Reference
p.Arg34fs * 60	Frameshift	-	0	N-Terminus	-	LOF	[27]
p.Val240Met	Missense	rs199473076	1.4 × 10^−5^	DI	Cytoplasmic S4-S5	GOF	[22]
p.Arg811Cys	Missense	rs794728864	0	DII	S4	-	-
p.Arg893His	Missense	rs199473172	4.0 × 10^−6^	DII	Pore	LOF	[25]
p.Arg1195His	Missense	rs199473596	2.5 × 10^−5^	Interdomain DII-DIII	Cytoplasmic	LOF/GOF	[27]
p.Asp1275Asn	Missense	rs137854618	8.0 × 10^−6^	DIII	S3	LOF	[24]
p.Trp1345_Ser1349delinsPhe	Inframe del/ins		0	DIII	S5	-	-
p.Arg1632Cys	Missense	rs878855292	4.0 × 10^−6^	DIV	S4	LOF/GOF	[28]
p.Arg1644His	Missense	rs199473282	4.0 × 10^−6^	DIV	Cytoplasmic S4-S5	GOF	[26]
p.Gly1661Arg	Missense	rs199473292	0	DIV	S5	LOF	[23]
p.Thr1708Asn	Missense	-	0	DIV	Pore	LOF/GOF	[25]
p.Ser1710Leu	Missense	rs137854604	1.6 × 10^−5^	DIV	Pore	LOF/GOF	[21]
p.Pro1730Leu	Missense	rs1060501142	8 × 10^−6^	DIV	Extracellular pore	-	-
p.Asp1741Glyfs * 48	Frameshift	rs1251085820	0	DIV S5/S6	-	-	-
p.Ala1778Asp	Missense	-	0	C-Terminus	Cytoplasmic	-	-
p.Leu1821fs * 10	Frameshift	rs794728924	0	C-Terminus	Cytoplasmic	LOF/GOF	[29]

* Functional interpretation according to functional studies expressing the variants in different heterologous systems. MAF: minor allele frequency; LOF: loss of function; GOF: gain of function.

**Table 2:** Clinical characteristics of *SCN5A*-channelopathy pediatric patients.

Case	Variant	Sex	Age of Onset	Syncope	Seizures	Device	NFCA	SCD	Arrhythmogenic Phenotype	SVT	VA	AVB	RBBB, LBBB	AS	MaxQTc (ms)
1	p.Ser1710Leu	M	14	-	-	PM	-	-	SSS, PCCD	aFL, CTI-D	-	3rd D	-	-	488 *
2	p.Ser1710Leu	M	2	Yes	-	PM	-	-	SSS	-	-	-	RBBB	-	682 *
3	p.Ser1710Leu	M	15	-	-	-	-	-	BrS	AF, aFL, CTI-D	-	1st D	-	-	413
4	p.Asp1275Asn	M	17	-	-	PM	-	-	SSS	-	-	3rd D	RBBB, LBBB	Yes	476 *
5	p.Arg893His	M	15	Yes	-	PM	-	-	SSS, VT	aFL, CTI-D	SMVT	3rd D	RBBB	Yes	542 *
6	p.Thr1708Asn	M	6	Yes	-	PM	-	-	SSS, PCCD, VT, BrS	AF, aFL, AT	SMVT	-	RBBB, LBBB	-	503 *
7	p.Thr1708Asn	M	10	-	-	PM	-	Yes	SSS, PCCD, VT	aFL	SMVT	1st D	RBBB, LBBB	-	481 *
8	p.Trp1345_Ser1349delinsPhe/ p.Pro1730Leu	F	3	Yes	-	PM	Yes	-	SSS, PCCD, VT, BrS	AF, aFL	SMVT, PVT	3rd D	RBBB, LBBB	Yes	495 *
9	p.Leu1821fs *10	M	12	-	-	PM	Yes	Yes	SSS, PCCD, VT	aFL	SMVT	2nd D	RBBB	-	550 *
10	p.Arg34fs *60/ p.Arg1195His	M	22mo	Yes	Yes	-.	Yes	Yes	VT, LQTS	-	SMVT, VF	-	-	-	519
11	p.Gly1661Arg	M	2	Yes	Yes	ICD	Yes	-	SSS, VT, BrS	-	SMVT, VF	-	RBBB	-	500 *
12	p.Arg893His	M	17	Yes	-	-	Yes	Yes	BrS	-	VF	-	-	-	402
13	p.Asp1741Glyfs *48/ p.Val240Met	M	4	Yes	-	PM	-	-	SSS, PCCD, VT	AF	SMVT	1st D	RBBB	-	630 *
14	p.Arg1644His	F	16	Yes	-	-	-	-	LQTS	AT	-	-	-	-	520
15	p.Arg811Cys	M	4	-	-	PM	-	-	SSS, PCCD, VT, BrS	aFL	SMVT	3rd D	RBBB, LBBB	Yes	519 *
16	p.Ala1778Asp	M	4	Yes	Yes	-	-	-	SSS, VT	AF	PVT, TdP	1st D	RBBB	Yes	529 *
17	p.Arg1632Cys	M	3	-	Yes	PM	-	Yes	SSS, PCCD, VT	AF	SMVT	-	RBBB, LBBB	-	462 *

NFCA: Non-fatal cardiac arrest; SCD: Sudden cardiac death; SVT: Supraventricular tachycardia; VA: Ventricular arrhythmia; AVB: Atrioventricular block; RBBB: Right bundle branch block; LBBB: Left bundle branch block; AS: Atrial standstill; MaxQTc: maximum corrected QT interval; SSS: Sick sinus syndrome; PCCD: Progressive cardiac conduction disease; VT: idiopathic ventricular tachycardia; BrS: Brugada syndrome; LQTS: long QT syndrome; PM: pacemaker; ICD: Implantable cardioverter defibrillator; AF: Atrial fibrillation; aFL: Atrial flutter; CTI-D: cavotricuspid isthmus-dependent atrial flutter. AT: atrial tachycardia; SMVT: Sustained monomorphic ventricular tachycardia; PVT: polymorphic ventricular tachycardia; FV: ventricular fibrillation; TdP: Torsade de points. * Prolonged QTc interval secondary to intraventricular conduction defect or AV block. Cases 9 and 10 were previously published [27,29].

## Data Availability

Data available on request from the corresponding authors, due to restrictions on patient privacy as established in the informed consent.

## References

[1] Veerman C.C., Wilde A.A., Lodder E.M. (2015). The cardiac sodium channel gene *SCN5A* and its gene product Na_V_1.5: Role in physiology and pathophysiology. Gene.

[2] Shy D., Gillet L., Abriel H. (2013). Cardiac sodium channel Na_V_1.5 distribution in myocytes via interacting proteins: The multiple pool model. Biochim. Biophys. Acta.

[3] Clatot J., Hoshi M., Wan X., Liu H., Jain A., Shinlapawittayatorn K., Marionneau C., Ficker E., Ha T., Deschenes I. (2017). Voltage-gated sodium channels assemble and gate as dimers. Nat. Commun..

[4] Remme C.A. (2013). Cardiac sodium channelopathy associated with *SCN5A* mutations: Electrophysiological, molecular and genetic aspects. J. Physiol..

[5] Ruan Y., Liu N., Priori S.G. (2009). Sodium channel mutations and arrhythmias. Nat. Rev. Cardiol..

[6] Wilde A.A.M., Amin A.S. (2018). Clinical Spectrum of *SCN5A* Mutations: Long QT Syndrome, Brugada Syndrome, and Cardiomyopathy. JACC Clin. Electrophysiol..

[7] Verkerk A.O., Amin A.S., Remme C.A. (2018). Disease Modifiers of Inherited *SCN5A* Channelopathy. Front. Cardiovasc. Med..

[8] Blich M., Khoury A., Suleiman M., Lorber A., Gepstein L., Boulous M. (2019). Specific Therapy Based on the Genotype in a Malignant Form of Long QT3, Carrying the V411M Mutation. Int. Heart J..

[9] Chockalingam P., Rammeloo L.A., Postema P.G., Hruda J., Clur S.A., Blom N.A., Wilde A.A. (2011). Fever-induced life-threatening arrhythmias in children harboring an *SCN5A* mutation. Pediatrics.

[10] Kilinc O.U., Tuzcu V. (2012). Successful elimination of significant arrhythmia burden with flecainide in an adolescent with long QT syndrome type 3. Congenit. Heart Dis..

[11] Kwon H.W., Lee S.Y., Kwon B.S., Kim G.B., Bae E.J., Kim W.H., Noh C.I., Cho S.I., Park S.S. (2012). Long QT syndrome and dilated cardiomyopathy with *SCN5A* p.R1193Q polymorphism: Cardioverter-defibrillator implantation at 27 months. Pacing Clin. Electrophysiol..

[12] Nijak A., Labro A.J., De Wilde H., Dewals W., Peigneur S., Tytgat J., Snyders D., Sieliwonczyk E., Simons E., Van Craenenbroeck E. (2020). Compound Heterozygous *SCN5A* Mutations in Severe Sodium Channelopathy with Brugada Syndrome: A Case Report. Front. Cardiovasc. Med..

[13] Sacilotto L., Epifanio H.B., Darrieux F.C., Wulkan F., Oliveira T.G., Hachul D.T., Pereira A.D., Scanavacca M.I. (2017). Compound Heterozygous *SCN5A* Mutations in a Toddler—Are they Associated with a More Severe Phenotype?. Arq. Bras. Cardiol..

[14] Tsukakoshi T., Lin L., Murakami T., Shiono J., Izumi I., Horigome H. (2018). Persistent QT Prolongation in a Child with Gitelman Syndrome and *SCN5A* H558R Polymorphism. Int. Heart J..

[15] Baruteau A.E., Kyndt F., Behr E.R., Vink A.S., Lachaud M., Joong A., Schott J.J., Horie M., Denjoy I., Crotti L. (2018). *SCN5A* mutations in 442 neonates and children: Genotype-phenotype correlation and identification of higher-risk subgroups. Eur. Heart J..

[16] De Ponti R., Marazzato J., Bagliani G., Leonelli F.M., Padeletti L. (2018). Sick Sinus Syndrome. Card. Electrophysiol. Clin..

[17] Baruteau A.E., Probst V., Abriel H. (2015). Inherited progressive cardiac conduction disorders. Curr. Opin. Cardiol..

[18] Brugada J., Campuzano O., Arbelo E., Sarquella-Brugada G., Brugada R. (2018). Present Status of Brugada Syndrome: JACC State-of-the-Art Review. J. Am. Coll. Cardiol..

[19] Schwartz P.J., Ackerman M.J. (2013). The long QT syndrome: A transatlantic clinical approach to diagnosis and therapy. Eur. Heart J..

[20] Li H., Durbin R. (2010). Fast and accurate long-read alignment with Burrows-Wheeler transform. Bioinformatics.

[21] Akai J., Makita N., Sakurada H., Shirai N., Ueda K., Kitabatake A., Nakazawa K., Kimura A., Hiraoka M. (2000). A novel *SCN5A* mutation associated with idiopathic ventricular fibrillation without typical ECG findings of Brugada syndrome. FEBS Lett..

[22] Fatima A., Kaifeng S., Dittmann S., Xu G., Gupta M.K., Linke M., Zechner U., Nguemo F., Milting H., Farr M. (2013). The disease-specific phenotype in cardiomyocytes derived from induced pluripotent stem cells of two long QT syndrome type 3 patients. PLoS ONE.

[23] Glazer A.M., Wada Y., Li B., Muhammad A., Kalash O.R., O’Neill M.J., Shields T., Hall L., Short L., Blair M.A. (2020). High-Throughput Reclassification of *SCN5A* Variants. Am. J. Hum. Genet..

[24] Gui J., Wang T., Jones R.P., Trump D., Zimmer T., Lei M. (2010). Multiple loss-of-function mechanisms contribute to *SCN5A*-related familial sick sinus syndrome. PLoS ONE.

[25] Kapplinger J.D., Giudicessi J.R., Ye D., Tester D.J., Callis T.E., Valdivia C.R., Makielski J.C., Wilde A.A., Ackerman M.J. (2015). Enhanced Classification of Brugada Syndrome-Associated and Long-QT Syndrome-Associated Genetic Variants in the *SCN5A*-Encoded Na(v)1.5 Cardiac Sodium Channel. Circ. Cardiovasc. Genet..

[26] Malan D., Zhang M., Stallmeyer B., Muller J., Fleischmann B.K., Schulze-Bahr E., Sasse P., Greber B. (2016). Human iPS cell model of type 3 long QT syndrome recapitulates drug-based phenotype correction. Basic Res. Cardiol..

[27] Medeiros-Domingo A., Tan B.H., Iturralde-Torres P., Tester D.J., Tusie-Luna T., Makielski J.C., Ackerman M.J. (2009). Unique mixed phenotype and unexpected functional effect revealed by novel compound heterozygosity mutations involving *SCN5A*. Heart Rhythm.

[28] Nakajima T., Kaneko Y., Saito A., Ota M., Iijima T., Kurabayashi M. (2015). Enhanced fast-inactivated state stability of cardiac sodium channels by a novel voltage sensor *SCN5A* mutation, R1632C, as a cause of atypical Brugada syndrome. Heart Rhythm.

[29] Tan B.H., Iturralde-Torres P., Medeiros-Domingo A., Nava S., Tester D.J., Valdivia C.R., Tusie-Luna T., Ackerman M.J., Makielski J.C. (2007). A novel C-terminal truncation *SCN5A* mutation from a patient with sick sinus syndrome, conduction disorder and ventricular tachycardia. Cardiovasc. Res..

[30] Ahmad F., McNally E.M., Ackerman M.J., Baty L.C., Day S.M., Kullo I.J., Madueme P.C., Maron M.S., Martinez M.W., Salberg L. (2019). Establishment of Specialized Clinical Cardiovascular Genetics Programs: Recognizing the Need and Meeting Standards: A Scientific Statement from the American Heart Association. Circ. Genom. Precis. Med..

[31] Kapplinger J.D., Tester D.J., Alders M., Benito B., Berthet M., Brugada J., Brugada P., Fressart V., Guerchicoff A., Harris-Kerr C. (2010). An international compendium of mutations in the *SCN5A*-encoded cardiac sodium channel in patients referred for Brugada syndrome genetic testing. Heart Rhythm.

[32] Abe K., Machida T., Sumitomo N., Yamamoto H., Ohkubo K., Watanabe I., Makiyama T., Fukae S., Kohno M., Harrell D.T. (2014). Sodium channelopathy underlying familial sick sinus syndrome with early onset and predominantly male characteristics. Circ. Arrhythm. Electrophysiol..

[33] Baskar S., Ackerman M.J., Clements D., Mayuga K.A., Aziz P.F. (2014). Compound heterozygous mutations in the *SCN5A*-encoded Nav1.5 cardiac sodium channel resulting in atrial standstill and His-Purkinje system disease. J. Pediatr..

[34] Bezzina C.R., Rook M.B., Groenewegen W.A., Herfst L.J., van der Wal A.C., Lam J., Jongsma H.J., Wilde A.A., Mannens M.M. (2003). Compound heterozygosity for mutations (W156X and R225W) in *SCN5A* associated with severe cardiac conduction disturbances and degenerative changes in the conduction system. Circ. Res..

[35] Cordeiro J.M., Barajas-Martinez H., Hong K., Burashnikov E., Pfeiffer R., Orsino A.M., Wu Y.S., Hu D., Brugada J., Brugada P. (2006). Compound heterozygous mutations P336L and I1660V in the human cardiac sodium channel associated with the Brugada syndrome. Circulation.

[36] Nunez L., Barana A., Amoros I., de la Fuente M.G., Dolz-Gaiton P., Gomez R., Rodriguez-Garcia I., Mosquera I., Monserrat L., Delpon E. (2013). p.D1690N Nav1.5 rescues p.G1748D mutation gating defects in a compound heterozygous Brugada syndrome patient. Heart Rhythm.

[37] Tan B.Y., Yong R.Y., Barajas-Martinez H., Dumaine R., Chew Y.X., Wasan P.S., Ching C.K., Ho K.L., Gan L.S., Morin N. (2016). A Brugada syndrome proband with compound heterozygote *SCN5A* mutations identified from a Chinese family in Singapore. Europace.

[38] Thongnak C., Limprasert P., Tangviriyapaiboon D., Silvilairat S., Puangpetch A., Pasomsub E., Sukasem C., Chantratita W. (2016). Exome Sequencing Identifies Compound Heterozygous Mutations in *SCN5A* Associated with Congenital Complete Heart Block in the Thai Population. Dis. Markers.

[39] Ghovanloo M.R., Atallah J., Escudero C.A., Ruben P.C. (2020). Biophysical Characterization of a Novel *SCN5A* Mutation Associated with an Atypical Phenotype of Atrial and Ventricular Arrhythmias and Sudden Death. Front. Physiol..

[40] Grandi E., Pandit S.V., Voigt N., Workman A.J., Dobrev D., Jalife J., Bers D.M. (2011). Human atrial action potential and Ca^2+^ model: Sinus rhythm and chronic atrial fibrillation. Circ. Res..

[41] O’Hara T., Virag L., Varro A., Rudy Y. (2011). Simulation of the undiseased human cardiac ventricular action potential: Model formulation and experimental validation. PLoS Comput. Biol..

[42] Marangoni S., Di Resta C., Rocchetti M., Barile L., Rizzetto R., Summa A., Severi S., Sommariva E., Pappone C., Ferrari M. (2011). A Brugada syndrome mutation (p.S216L) and its modulation by p.H558R polymorphism: Standard and dynamic characterization. Cardiovasc. Res..

[43] Viswanathan P.C., Benson D.W., Balser J.R. (2003). A common *SCN5A* polymorphism modulates the biophysical effects of an *SCN5A* mutation. J. Clin. Investig..

[44] Ye B., Valdivia C.R., Ackerman M.J., Makielski J.C. (2003). A common human *SCN5A* polymorphism modifies expression of an arrhythmia causing mutation. Physiol. Genom..

[45] Nijak A., Saenen J., Labro A.J., Schepers D., Loeys B.L., Alaerts M. (2021). iPSC-Cardiomyocyte Models of Brugada Syndrome-Achievements, Challenges and Future Perspectives. Int. J. Mol. Sci..

[46] Watanabe H., Yang T., Stroud D.M., Lowe J.S., Harris L., Atack T.C., Wang D.W., Hipkens S.B., Leake B., Hall L. (2011). Striking In vivo phenotype of a disease-associated human *SCN5A* mutation producing minimal changes in vitro. Circulation.

[47] Kamga M.V.K., Reppel M., Hescheler J., Nguemo F. (2021). Modeling genetic cardiac channelopathies using induced pluripotent stem cells—Status quo from an electrophysiological perspective. Biochem. Pharmacol..

